# Research on collapse ultimate load of fabricated reinforced concrete column and steel beam composite frame structure

**DOI:** 10.1038/s41598-022-17936-z

**Published:** 2022-08-10

**Authors:** Youquan Liu, Jingang Xiong, Jiancong Wen, Moqiang Xiong

**Affiliations:** 1 School of Infrastructure Engineering, Nanchang University, Nanchang, 330031 Jiangxi Province China; 2 Jiangxi Provincial Engineering Laboratory of Nearly Zero Energy Building, Nanchang, China

**Keywords:** Civil engineering, Engineering

## Abstract

In this work, the collapse ultimate load of a prefabricated reinforced concrete column–steel beam composite frame structure was studied. During the study, the “new RCS beam-to-column joint” was used as the beam-to-column connection in the experimental model. Further, the half-scale fabricated RCS space frame structure (2-story, 1 × 2 bay) was subjected to instantaneous failure experiments twice at the bottom of the side column under various load levels and the 2A column was quickly pulled out by the traction force of the vehicle. The experimental results demonstrated that the method of dismantling the failure column provided a relatively true response to the condition of progressive collapse. The remaining RCS structure was found to be in the elastic stage during various load level tests. Moreover, the displacement time history curve did not have a vibration phenomenon during the first experimentation. The SAP2000 finite element program was used to verify that the test results were similar to that of the numerical simulation results, and it was further explored and found that the collapse ultimate load value was 10.25 times the structure design load value.

## Introduction

The prefabricated reinforced concrete (RC) column–steel (S) beam composite frame structure is abbreviated as the RCS composite structure, which refers to the frame structure prefabricated using a precast steel beam, reinforced concrete column, and the composite floor slab on the construction site. On comparison with the steel column, the concrete column of this new type of composite structure has better compression resistance, greater rigidity, durability, and fire resistance. Moreover, it saves steel and improves the stability of the structure. Further, when compared with the reinforced concrete beam, the steel beam has good flexural performance, lightweight, convenient construction, reduces the component section size, and increases the effective space for use^1^. Therefore, prefabricated RCS composite frame structure is one of the leading structural systems conforming to the development trend of building industrialization, with a broad development prospect.

Due to unexpected accidents, the partial failure of the building structure causes chain failure of the components and leads to the collapse of most of the structure or the whole structure. This is called a progressive collapse. If the building structure collapses, it is bound to cause serious casualties and huge economic losses. Moreover, the progressive collapse behavior of the prefabricated RCS composite frame structure is different from that of the cast-in-place concrete frame structure or the steel frame structure. Therefore, it is necessary to study the progressive collapse resistance performance of the prefabricated RCS composite frame structure.

Shiekh and Deierlein et al. (1989) conducted testing of the low-cyclic experiments under repeated loading conditions on seventeen joint specimens with RCS intermediate layer and studied the influence of the joint failure mode, strength, stiffness, and the structural measures on the joint performance. Based on the study, the analytical formula to obtain the joint shear strength was developed^2,3^. Kanno and Deierlein (1993) conducted low-cyclic experiments under repeated loading conditions, and the capacity differences during the deformation, bearing, and energy dissipation of the specimens under various failure mechanisms were studied^4,5^. Parra-Montesinos and Weight (2000) studied the seismic performance of the edge joint in the middle layer of the RCS composite frame under cyclic loading conditions^6^. Liang and Parra-Montesinos (2004) carried out experiments on four RCS spatial joints under low cyclic loading conditions in order to study the hysteretic behavior, story drift, and joint deformations^7^. Chou et al. (2010) conducted a series of “column through type” RCS composite frame structure (full-scale, single-story, two-bay) seismic performance experiments, and verified whether the connection form was feasible. Further, they studied the seismic response under different load modes^8^. Azar et al. (2013) used Open Sees for nonlinear static analysis in order to simulate the influence of the nodes on the overall behavior of the RCS composite frame structure^9^. The results showed that RCS joints could increase the lateral bearing capacity of the whole frame. Moreover, by using a steel beam instead of the reinforced concrete beam, the overall performance was found to be significantly improved.

In 1968, the collapse of the Ronan Point apartment in the United Kingdom made the architecture experts to pay close attention to the progressive collapse. In 1970, the United Kingdom included the progressive collapse resistance to the building code. In 1975, Canada added a clause to prevent structural collapse in the building code. Other countries, including Sweden, Denmark, and the Netherlands, have also added the progressive collapse provisions in the code. Then, Japan (2005) and other countries also compiled the specifications of structural progressive collapse^10^. Thereafter, the progressive collapses caught the attention of Americans after the events of the Murrah Federal Building and the World Trade Center (2005)^11–14^. Although these regulations emphasize the importance and harm of the progressive collapse to the structure, some regulations are not specific enough and vague in meaning, which is inconvenient for practical operations.

In this work, the “new RCS beam-to-column joint”^15^ was applied to the beam-to-column connection, and a half-scale prefabricated RCS space frame structure (1 × 2-bay, 2-story) was manufactured as the experimental model. In order to explore the collapse ultimate load, the experimental model was subjected to the column instantaneous failure twice under various levels of loads. The weakened column (2A) was rapidly pulled out by the traction force of the vehicle, and the displacement and strain at the important positions were recorded using the dynamic data acquisition instrument. The method of the failure column demolition that was used in this work was found to be safer and easier to operate than the hydrogen gas-gun impact. Further, the hydrogen gas-gun impact method, which was also used by Kunnath et al. was employed in this work^16^.

In this work, the collapse ultimate load value of the fabricated RCS composite frame structure was verified and was found to be 10.25 times the structure design load value. The experimental results also revealed that the dismantling method of the failure column is safe and feasible.

## Experimental methods

The experimental methods include the model selection, “New RCS beam-to-column joint” model, experimental prototype and model, experimental, loading, failure column removal, and the data test scheme. They are briefly described below.

### Selection of the model

The “new RCS beam-to-column joint”^15^ that was developed by our research group was applied to the beam-to-column connection of the prototype structure, and the prototype structure was designed as per the requirements of the Chinese building Codes^17–19^. The experimental model was taken from A to B span and 1 to 2-story of the prototype structure, and it was scaled down to 1/2.

### “New RCS beam-to-column joint” model

**Figure 1:**
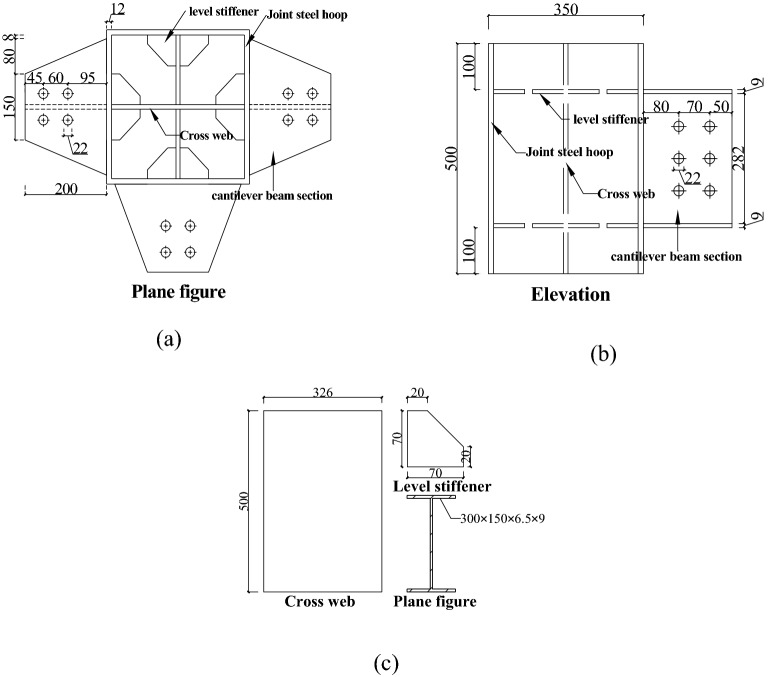
Constructional details of the “new RCS beam-to-column connection”.

**Figure 2:**
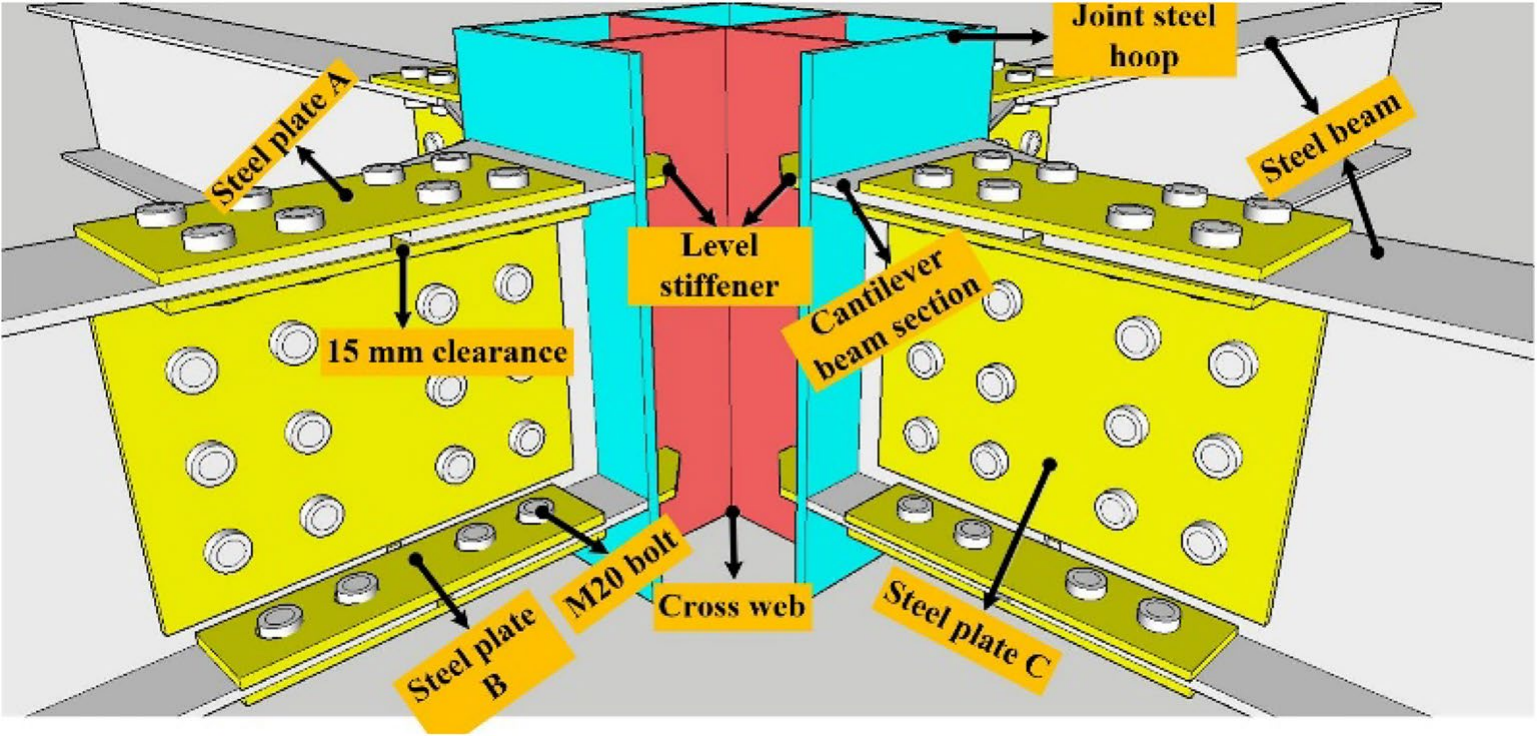
Connection diagram of the “new RCS beam-to-column connection”.

The “new RCS beam-to-column joint”^15^ includes the joint steel hoop, cross-web, level stiffener, and cantilever beam section. The specification of the joint steel hoop was 350 mm × 350 mm × 500 mm × 12 mm. The cross-web and horizontal stiffeners were welded inside it, and the cantilever beam of length 2000 mm was welded outside it. The “new RCS beam-to-column joint”^15^ specifications are shown in Table 1, and the construction and connection are shown in Figs. 1 and 2, respectively.

**Table 1:** Specifications of the “new RCS beam-to-column connection”.

Name	Size (mm)	Height/thickness/length (mm)
Joint steel hoop	350 × 350 × 12	Height: 500
Cross web	500 × 326	Thickness: 10
Level stiffener	20 × 70	Thickness: 9
Cantilever beam section	300 × 150 × 6.5 × 9	Length: 200
Steel plate A	315 × 150	Thickness: 8
Steel plate B	315 × 70	Thickness: 8
Steel plate C	355 × 240	Thickness: 8

### Experimental prototype

**Figure 3:**
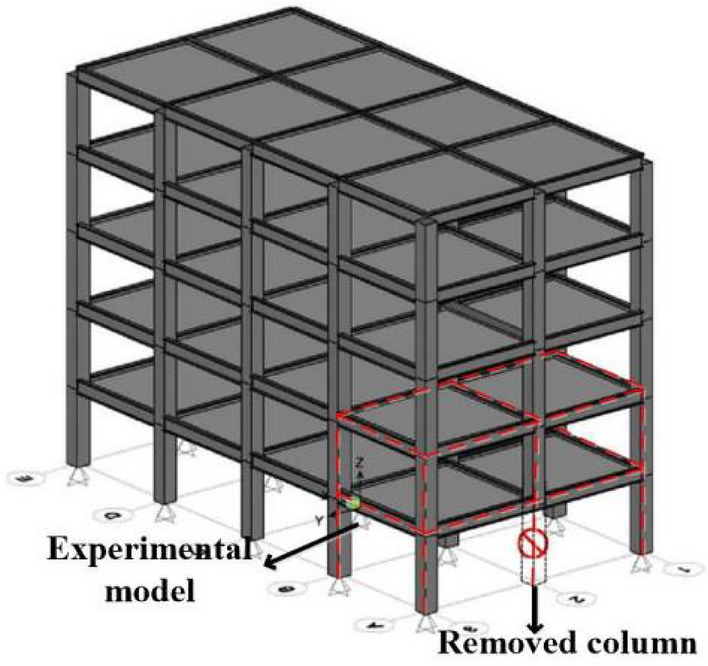
Perspective view.

**Figure 4:**
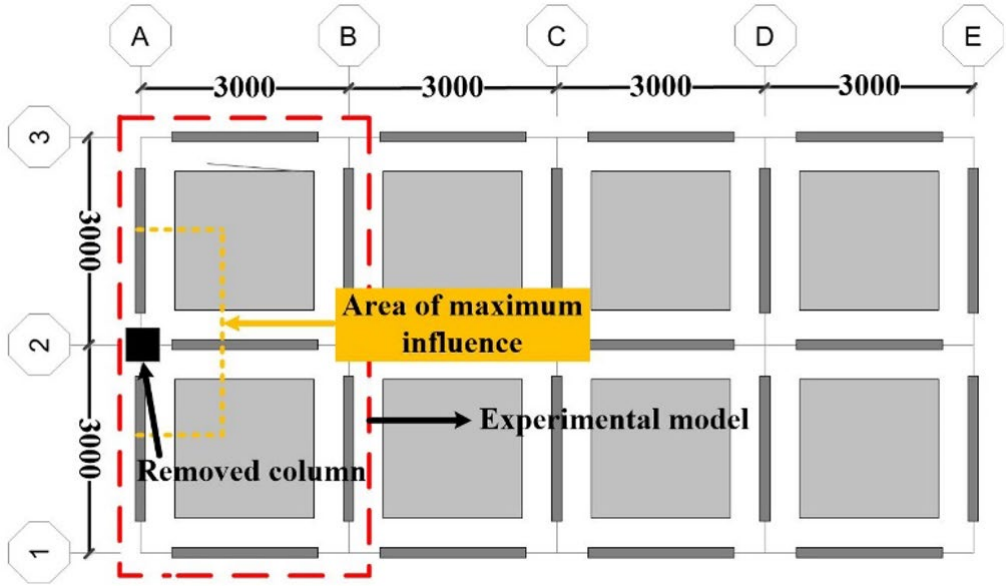
Plan view.

In this paper, the prefabricated RCS structure prototype (3 × 4-bay, 5-story) was designed as per the requirements of the Chinese Building Codes^17–19^. The C40 (HRB335, Q345) was used for the concrete (rebar, steel member). The cross-sectional size of the steel beam (concrete column, open trough steel sheeting composite floor) was 600 mm × 300 mm × 13 mm × 18 mm (700 mm × 700 mm, 80 mm). The seismic design intensity of the structure prototype was 7-degree^18^, whose peak ground acceleration (PGA) corresponding to the exceeding probability of 10% in 50 years was 0.1 g, in which g refers to the acceleration of gravity. The perspective view and plan view are shown in Figs. 3 and 4.

### Experimental model

**Figure 5:**
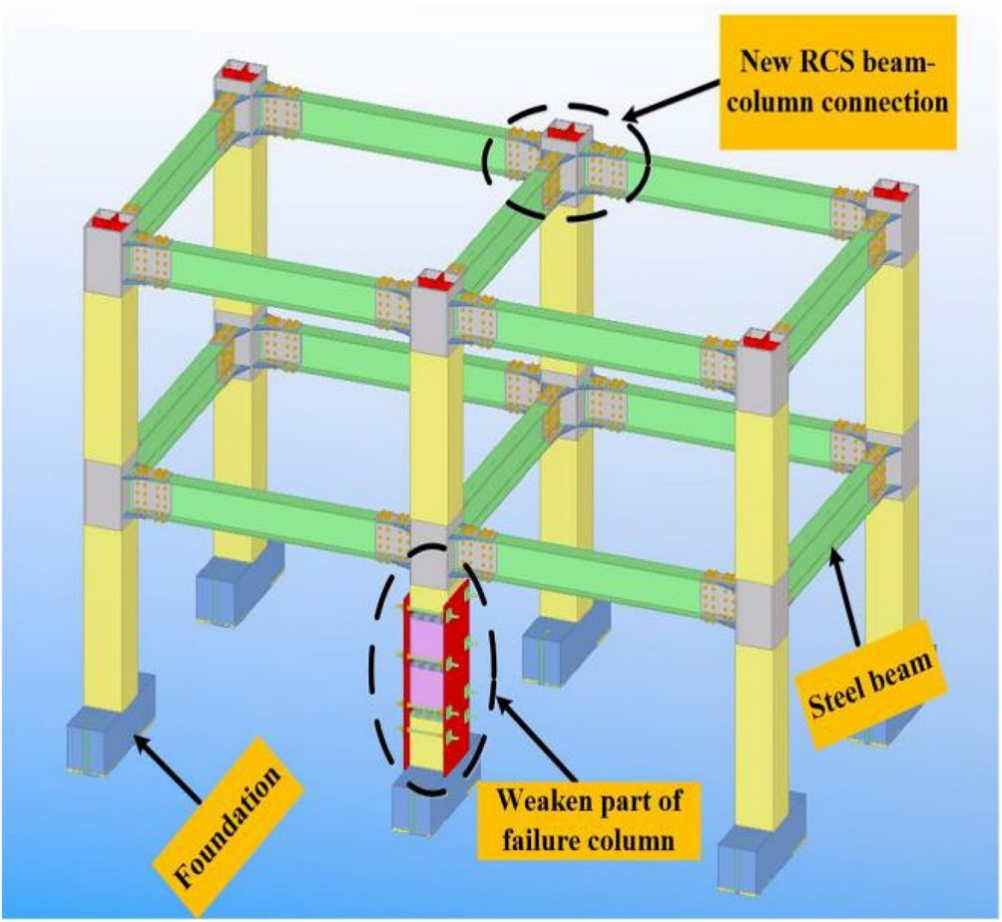
Experimental model.

**Figure 6:**
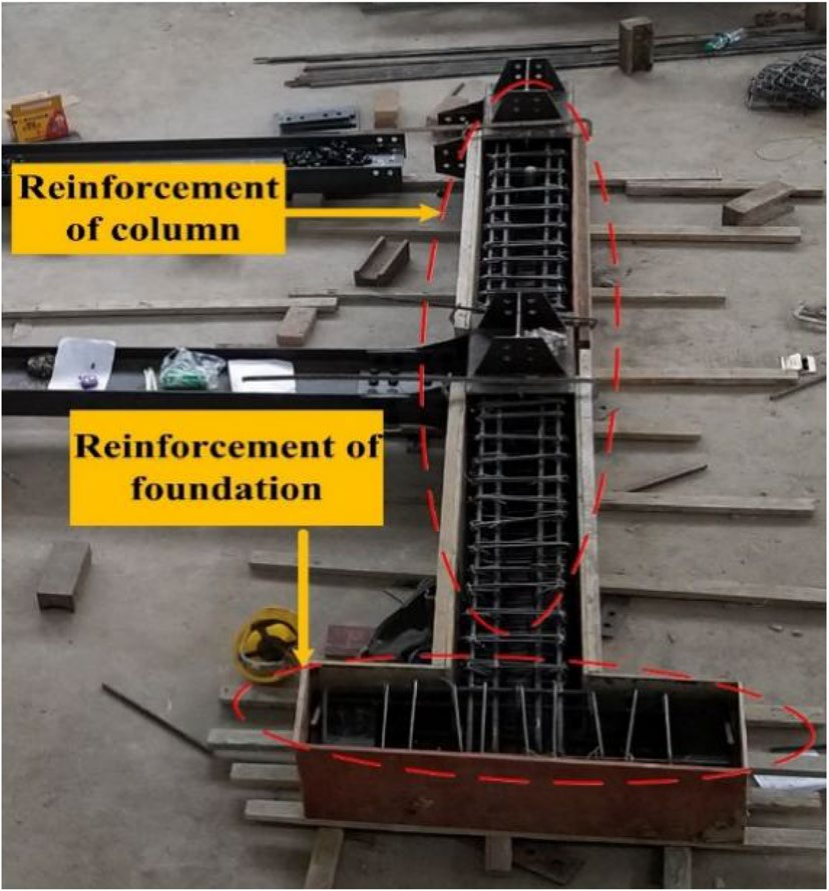
Photograph of the scene reinforcement.

**Figure 7:**
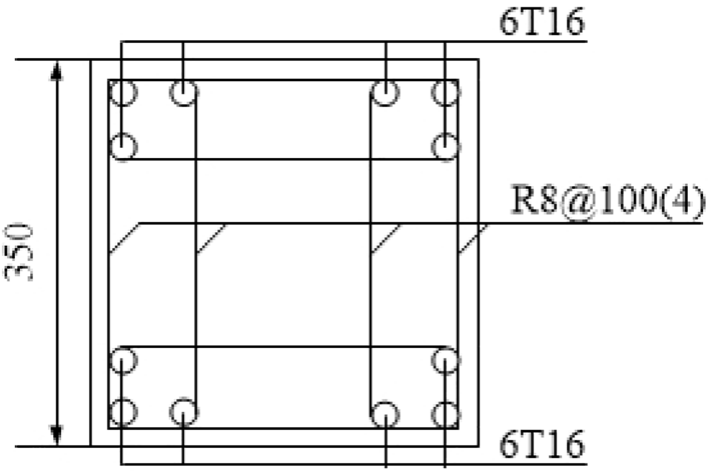
Column reinforcement.

**Figure 8:**
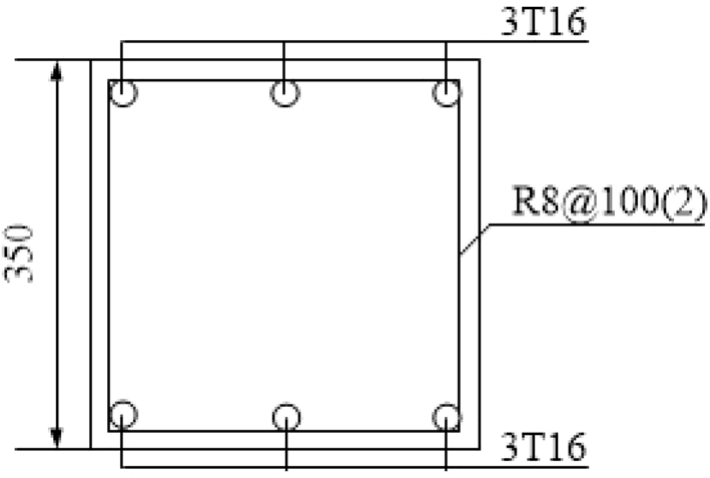
Foundation reinforcement.

The experimental model was taken from A to B span and 1 to 2-story of the prototype structure, and it was scaled down to half. The membrane effect of the slab was not considered in the experimental model and the cross-sectional size of the steel beam (concrete column) was taken as 300 mm × 150 mm × 6.5 mm × 9 mm (350 mm × 350 mm). Further, the experimental model was 3 m span in both the X and Y directions, and the first (second) storey of the experimental model was 2 m (1.8 m) in height. Moreover, the foundation was replaced using a ground beam. The same batch of the concrete material characteristic was tested, and the mean compressive strength (*f*_*cu*_) was found to be 39 MPa. The experimental model is shown in Fig. 5, and the photographs of the scene reinforcement, the reinforcement of column, and foundation are shown in Figs. 6, 7 and 8.

### Experimental scheme

In order to explore the collapse ultimate load of the prefabricated RCS structure, the test model was subjected to the instantaneous failure experiments twice under various levels of loads. The failure column was constrained vertically after the remaining RCS structure was stationary, and then the load was increased during the second experimentation.

### Loading scheme

The first experimental load was the 2.5 layers design load from the most influential area of the prototype structure. After the first experimentation, it was found that the remaining RCS structural had lesser deformation, and was in the early elastic stage. Moreover, the 2.5 layers design load was far less than the collapse ultimate load value. In order to explore the collapse ultimate load, the load was increased based on the first experimentation, and the second experimental load was taken as the 5 layers design load in the most affected area of the prototype structure. Due to the limited loading space of the experimental model, the load could not be added to continue the experimentation after the second experimentation. However, in order to explore the collapse ultimate load value, the SAP2000 finite element program was used for the simulation analysis in this work.

**Figure 9:**
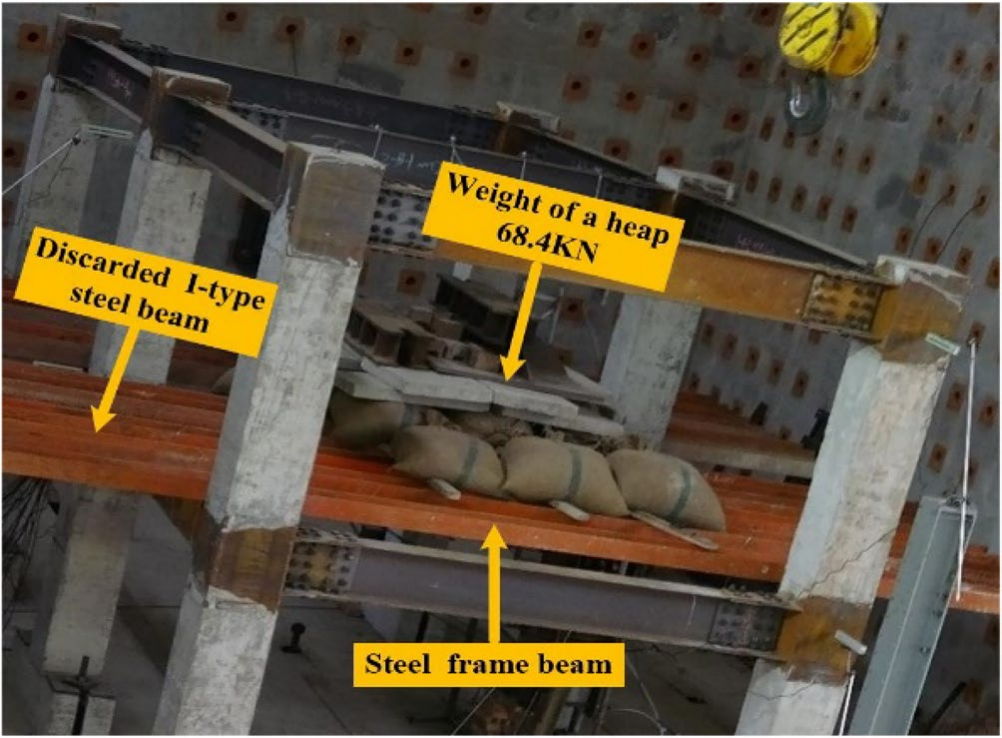
First experimental load.

**Figure 10:**
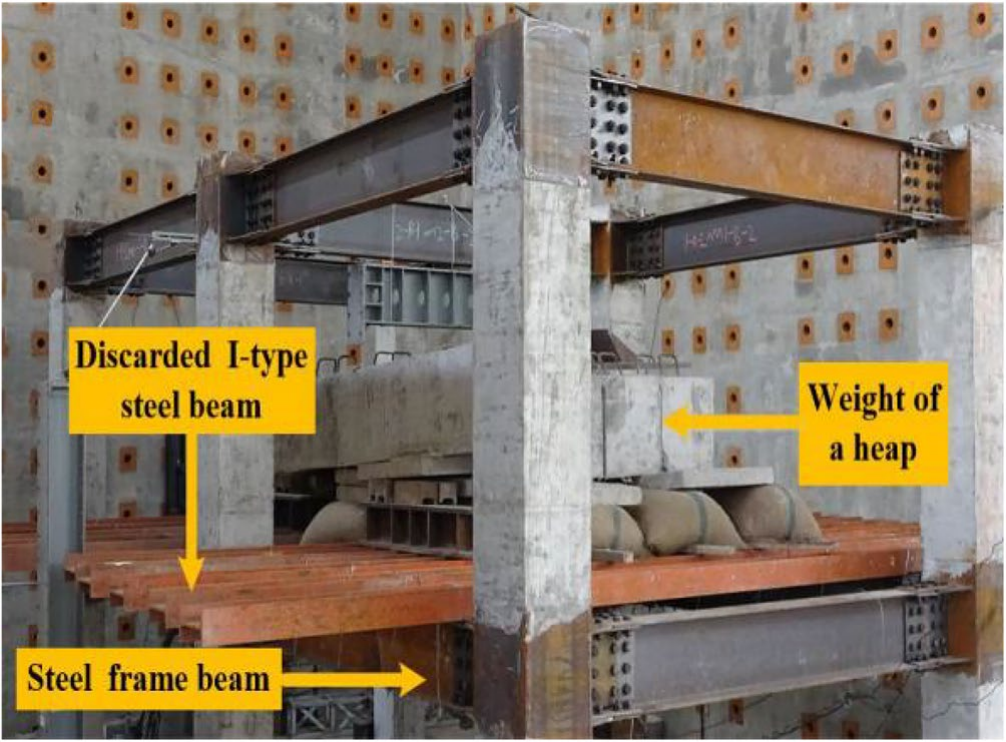
Second experimental load.

During the first experimentation, the applied load was 68.4 kN. When the remaining RCS structure was stationary, the steel columns and several pieces of thin steel plates were used to constrain the failure columns vertically. The first experimental load is shown in Fig. 9. During the second experimentation, the applied load was 140.4 kN, and the second experimentation load is shown in Fig. 10.

### Failure column removal scheme

**Figure 11:**
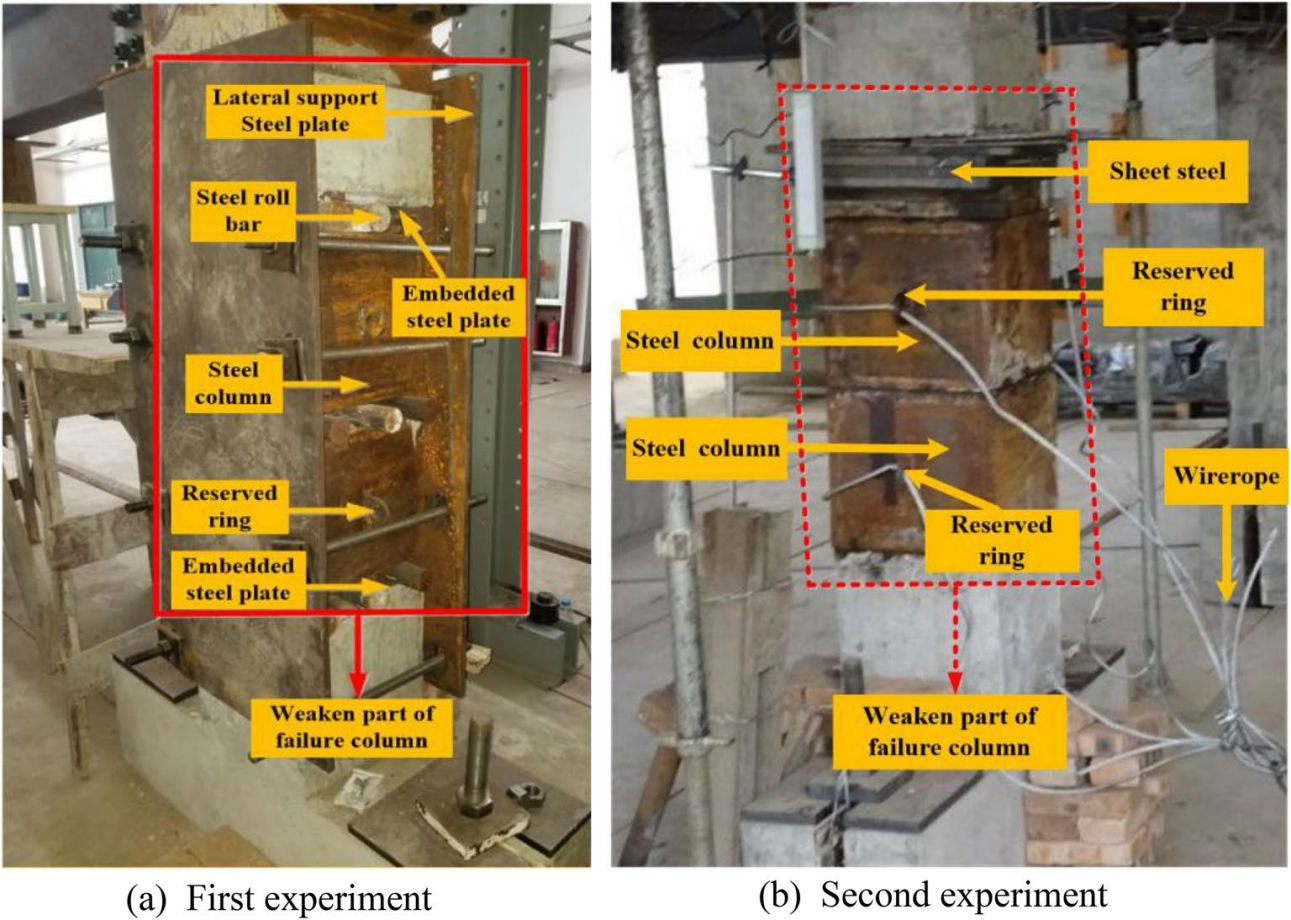
Photographs of the failure column.

During the first experimentation, the weaker part of the failure column included the steel column, steel roll bar, and the embedded steel plate from bottom to top. The photograph of the failure column is shown in Fig. 11a. The two steel columns and several pieces of thin steel plates were used to constrain the failure column vertically after the remaining RCS structure was stationary.

During the second experimentation, the weaker part of the failure column included the sheet steel, steel column, and the embedded steel plate from top to bottom. The photograph of the failure column is shown in Fig. 11b. It was observed that the event of accidental impact is one of the conditions that cause progressive collapses. In order to have a clear idea about the accidental crash events, the weaker part of the failure column was pulled out quickly using a vehicle traction force. Further, one end of the wire rope was fixed on the reserved rings, and the opposite end of the rope was attached to the car tow hook. The method of demolition of the failed column during the second experimentation was found to be the same as that of the first experimentation.

### Date test scheme

**Figure 12:**
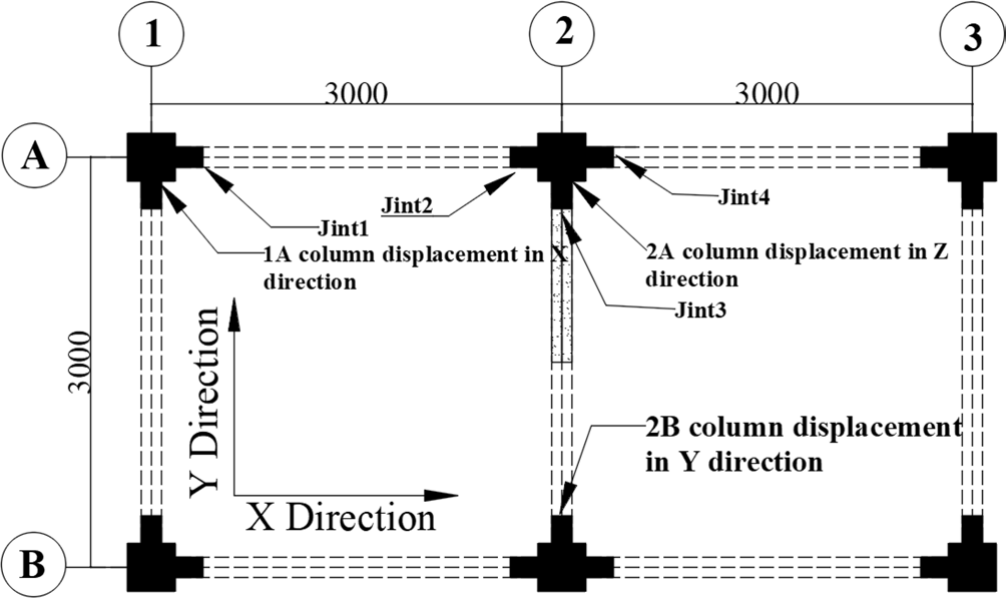
Displacement and strain test points.

**Figure 13:**
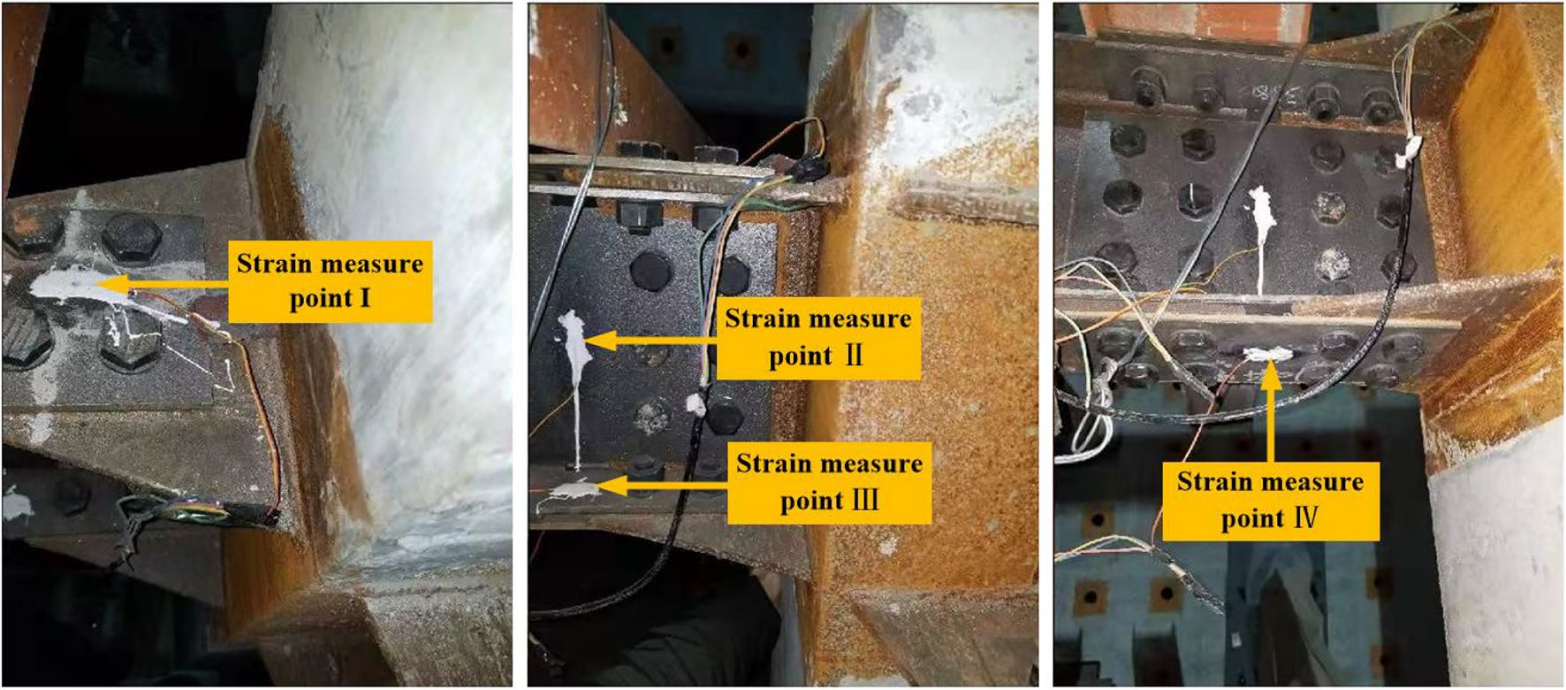
Strain distribution.

In order to meet the requirements of the data acquisition, the dynamic data acquisition instrument was used to record the displacements and strains. The displacement sensors were set in the Z direction of column 2A top and the X (Y) direction of column 1A (2B). Four beam-to-column joints were selected and named Joint 1, Joint 2, Joint 3, and Joint 4. The upper flange steel plate A, web steel plate C, lower flange steel plate A and B were set as the strain measurement points in each joint, and they were named as I, II, III, IV, respectively. The displacement and strain test points are shown in Fig. 12, and the strain distribution is shown in Fig. 13.

## Experimental results

The experimental results of the first and second experimentations are discussed below.

### Results and analysis of the first experimentation

**Figure 14:**
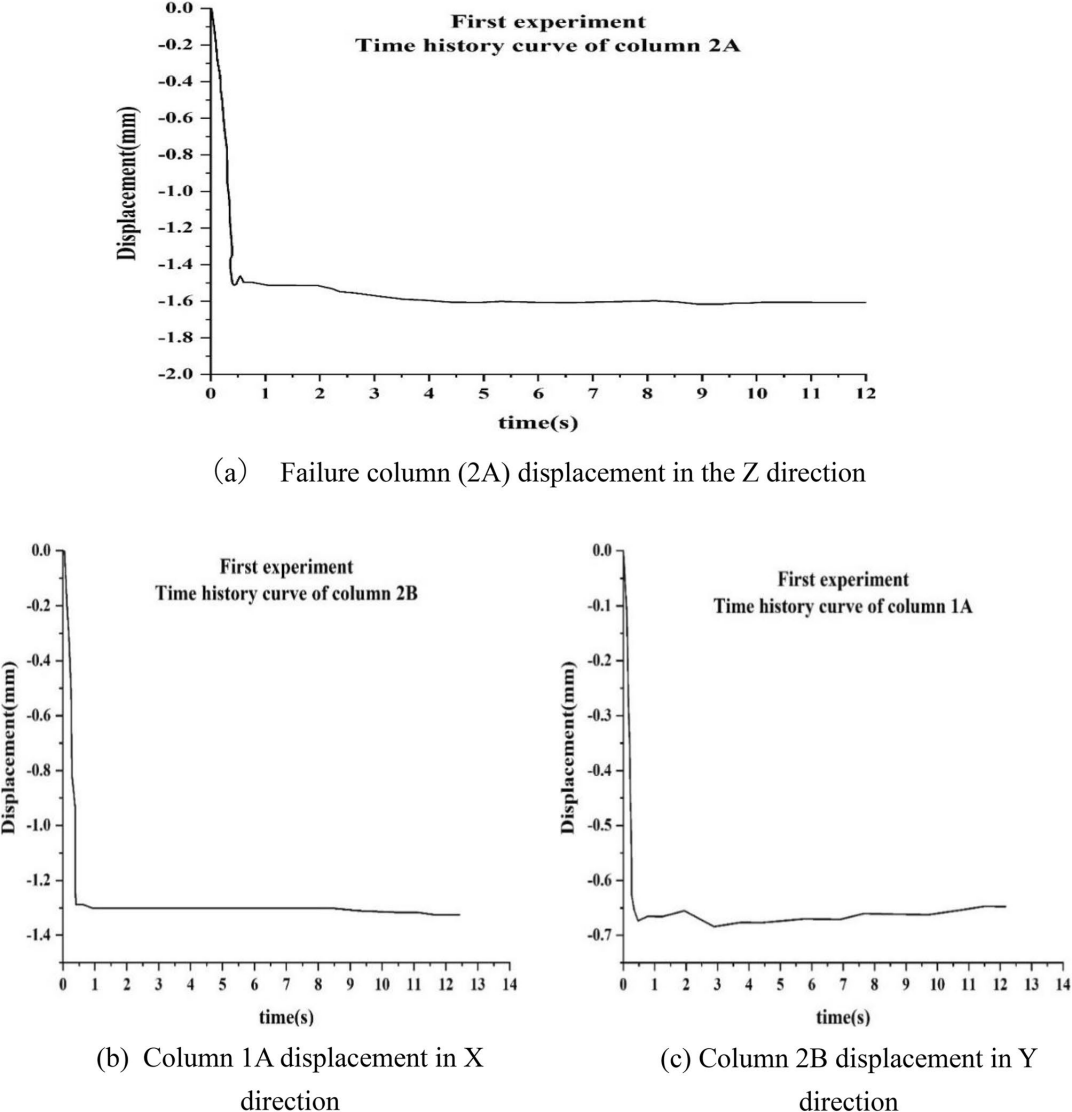
Time history curves of displacement in the first experimental.

From Fig. 14a,b,c, it is observed that the vertical displacement of the top of the failure column (2A) rapidly reaches 1.52 mm within 0.26 s when the weaker part was pulled out, and thereafter the displacement becomes stable at 1.64 mm. The displacement of column A1 rapidly is found to reach 0.67 mm within 0.46 s and rebound slightly between 0.46 and 1.94 s. Then, the displacement tends to become stable at 0.67 mm. The displacement of column B2 in the Y direction is found to reach 1.28 mm within 0.40 s, and then the displacement tends to become stable at 1.3 mm. When compared with the cast-in-place concrete structure, the prefabricated RCS composite frame structure is found to have no vibration phenomenon when the load was small. This may be due to the reason that the vibration needs to overcome the work done by the bolt slippage and consume vibration energy. Therefore, the time history curve of the displacement did not fluctuate up and down.

It is also observed that the remaining RCS structure did not enter the stable state immediately after reaching the instantaneous maximum displacement, but entered the slow development state. Moreover, when the remaining RCS structure was stable, the two steel columns and several sheets of steel were used to constrain the vertical direction of the A2 column.

**Figure 15:**
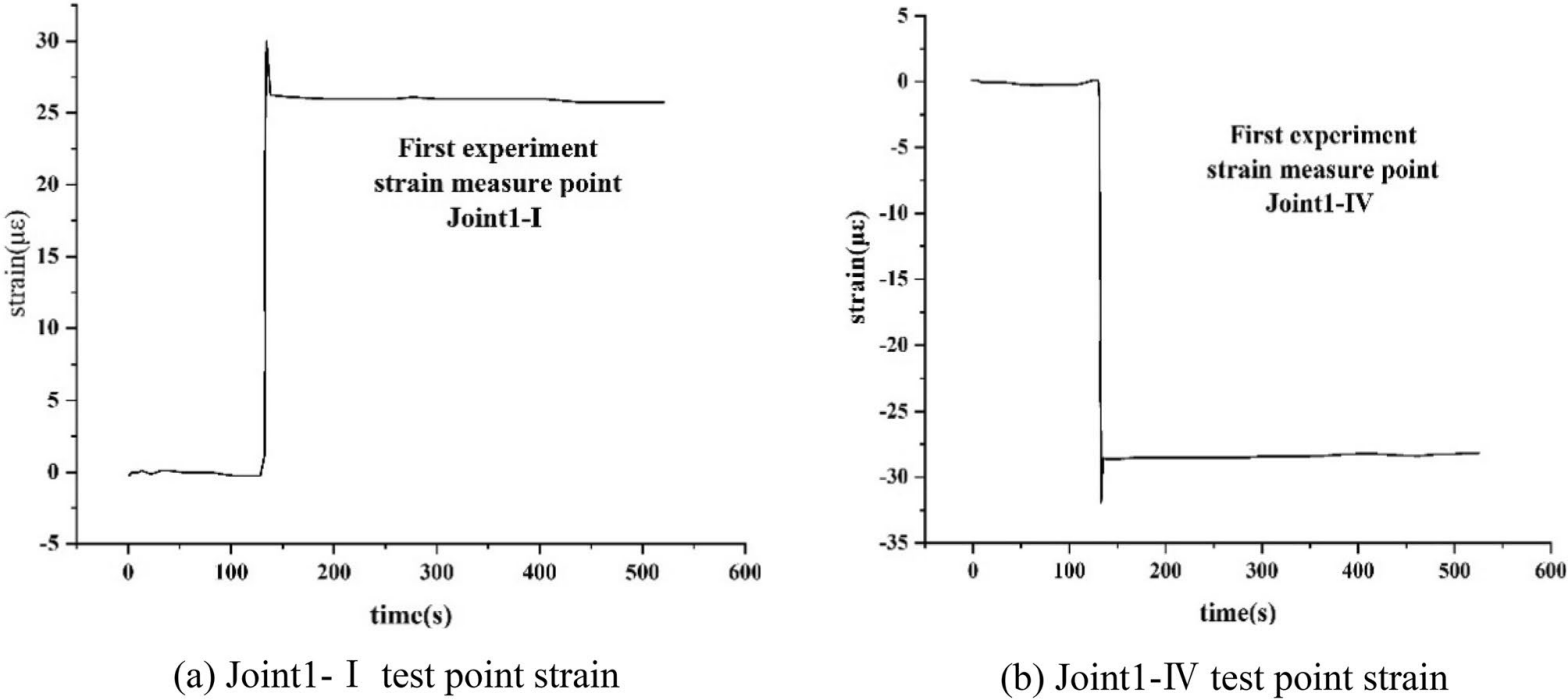
Time history curves of strain in the first experimentation.

From Fig. 15a,b, it is observed that the time history curve of strain and displacement are similar and both reach the maximum immediately. The tensile strain is found to reach 29.98 με at Joint 1-I, and then recovered to 26.2 με and became stable. The compressive strain reached − 31.9 με at Joint 1-IV, and then was recovered to − 28.6 με and became stable, where με refers to the unit symbol of microstrain. During the first experimentation, the change of the other 14 strains is found to be less, and therefore it was neglected in this work.

### Results and analysis of the second experimentation

**Figure 16:**
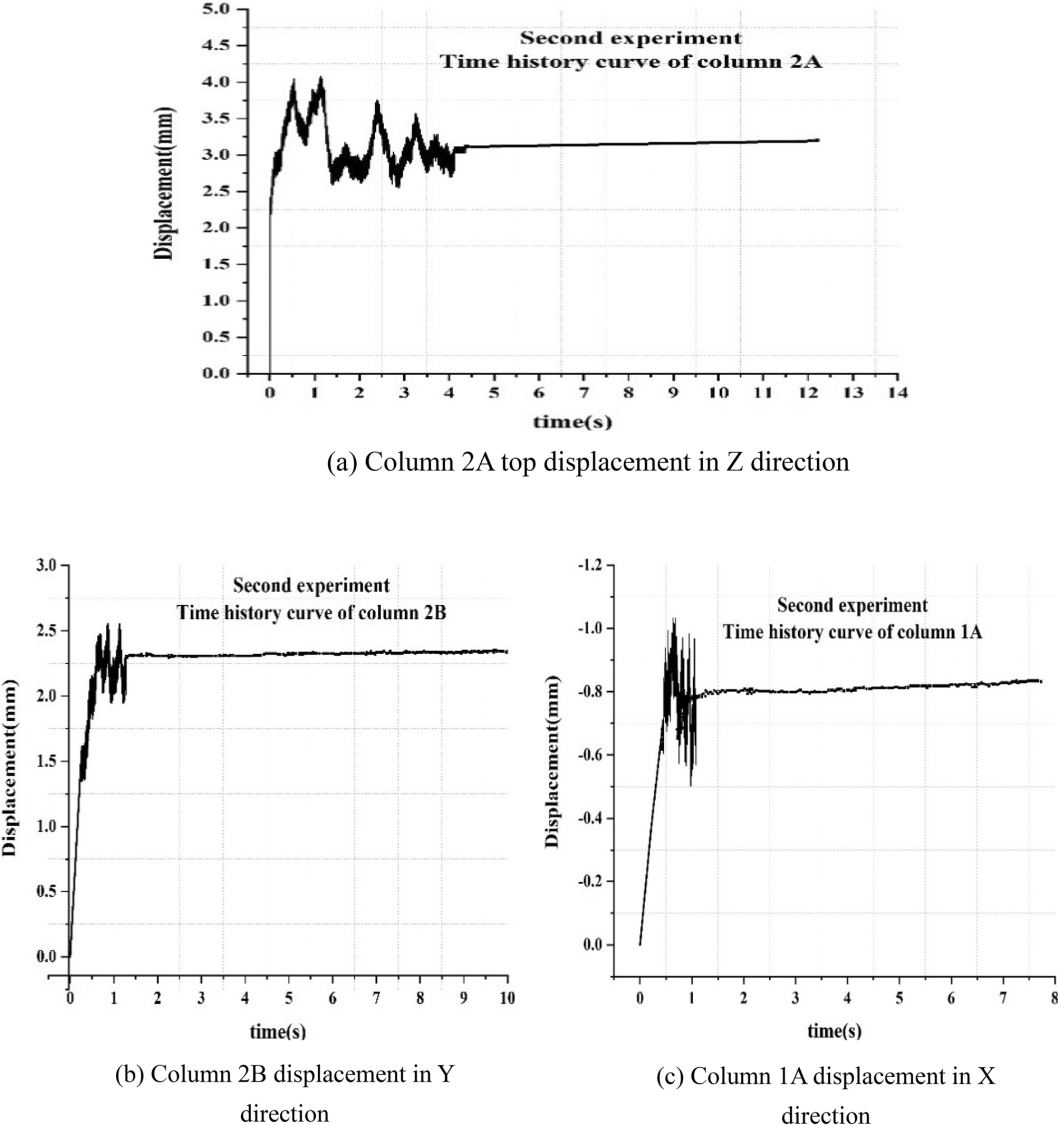
Time history curves of displacement in the second experimentation.

The top displacement of the column 2A in the Z direction is shown in Fig. 16a. From the figure, it is observed that as the screw diameter is 2 mm smaller than that of the bolt hole diameter, the Z-direction displacement is found to reach 2.255 mm during the period from 0 to 0.02 s. The contact time is found to be 0.02 to 0.533 s between the bolt and the inner wall of the hole. Further, the displacement in the Z direction is observed to increase more slowly than that in the previous 0.02 s. The Z direction vibration is found to appear in 0.53 to 4.2 s, and it entered a stable development stage after 4.16 s.

The displacement of the column 2B in the Y direction is shown in Fig. 16b. From the figure, it is observed that the initial response of column 2A is ahead of column 2B about 0.146 s. Further, it increases to 2.49 mm within 0.67 s, and the Y direction swings between 0.68 and 1.26 s, with a swing peak of 2.60 mm. The swing duration of column 2A in the Z direction was found to be longer than that of column 2B in the Y direction. Further, when the vibration is transmitted to the 2B column joint, it was necessary to overcome the work done by the bolt slippage and consumes the vibration energy.

The displacement of column 1A is shown in Fig. 16c. From the figure, it is observed that it increases to 1.012 mm from 0 to 0.683 s, and then swings between 0.678 and 1.069 s. Finally, it enters a stable growth stage after 1.071 s.

**Figure 17:**
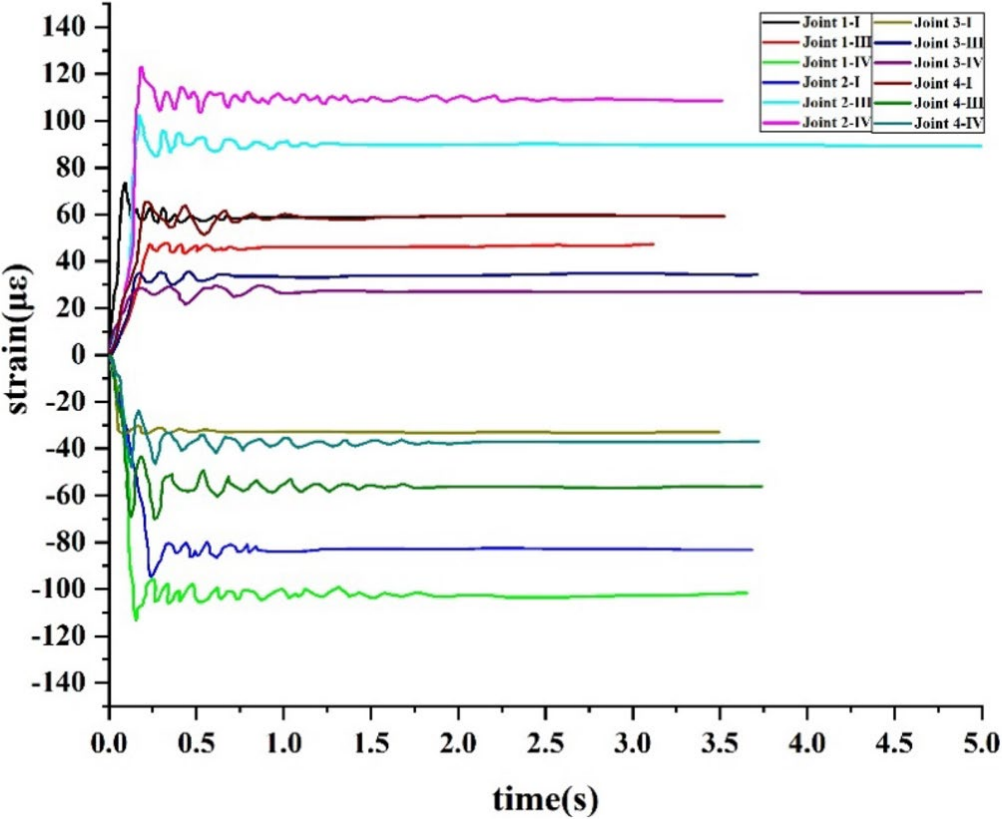
Time history curves of strain in second experimental.

The time history curves of the strain in the second experimentation are shown in Fig. 17. From the figure, it is observed that the maximum compressive strain at Joint 2-I is found to be 94.22 με and tends to 83 με after stabilization. The maximum pull strain at Joint 2-III is observed to be 103.09 με and tends to 89.53 με after stabilization. The maximum pull strain at Joint 2-IV is 122.9 με and tends to 109.13 με after stabilization. Further, when Joint 1-I test point is pulled, and Join 1-III and Join 1-IV test points were compressed, the maximum compressive strain at Joint 1-IV is found to be 110.84 με and tends to 102.6 με after stabilization. Then, Joint 3-I test point was compressed, and Joint 3-III and Joint 3-IV test points were pulled. Thereafter, Joint 4-I test point was pulled, and Joint 4-III and Join 4-IV test points were compressed. The strain of Joint 1, 2, 3, and 4 was far less than the yield strain and all of them were found to be in the early elastic stage, and the remaining RCS structure was also in the elastic stage.

It was also observed that the shear strains at Joint 1-II, Joint 2-II, Joint 3-II, and Joint 4-II did not change during the test. According to the study, the steel plate C could not be transferred using a shear force when the axial force of column 2A was transferred to the beam-to-column joint, however, it could be transferred using a friction force of the bolts on the web.

## Numerical analysis

The numerical analysis of the first and second experimentation are discussed below.

### First experimental numerical analysis

**Figure 18:**
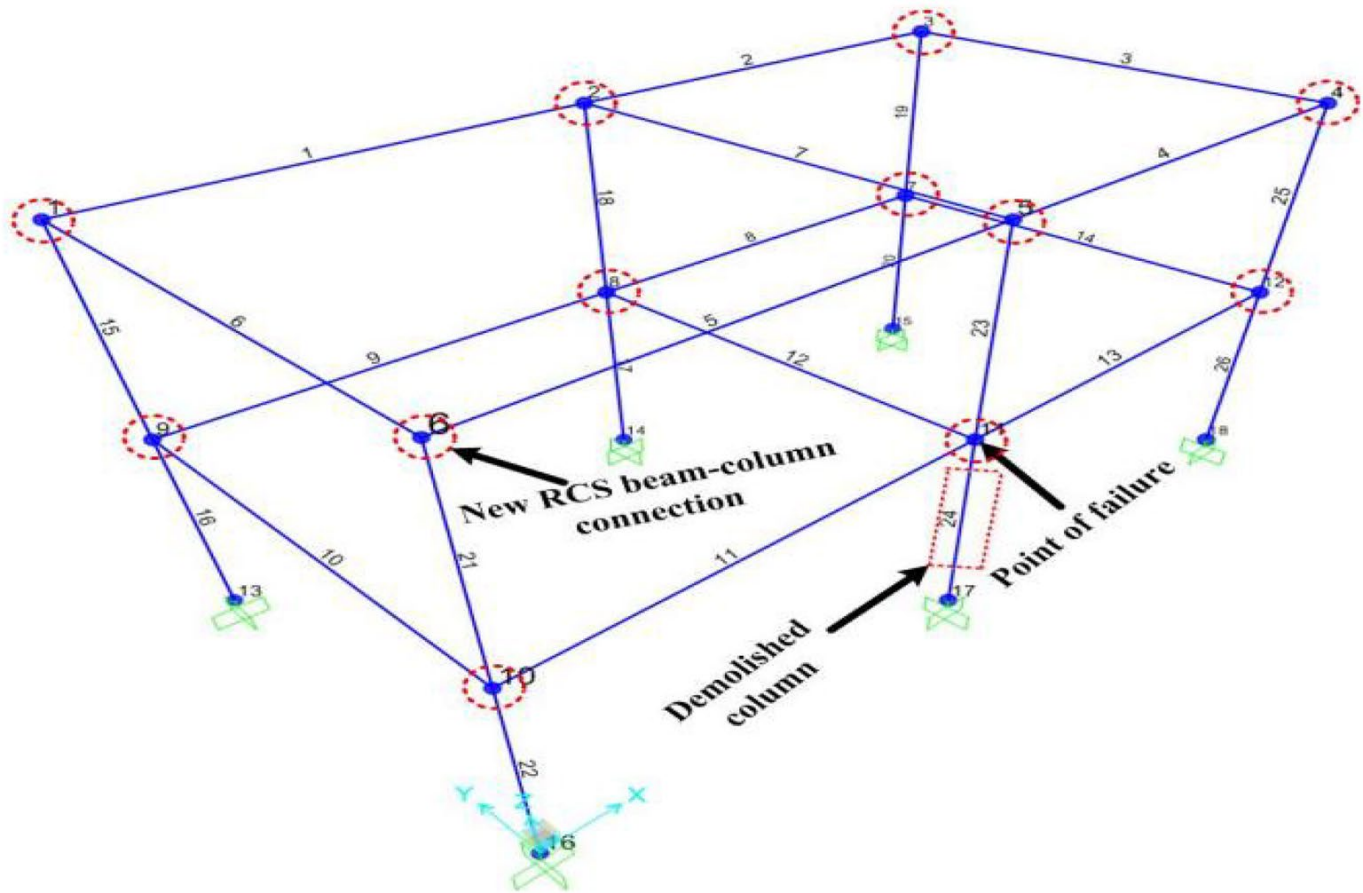
Finite element model.

**Figure 19:**
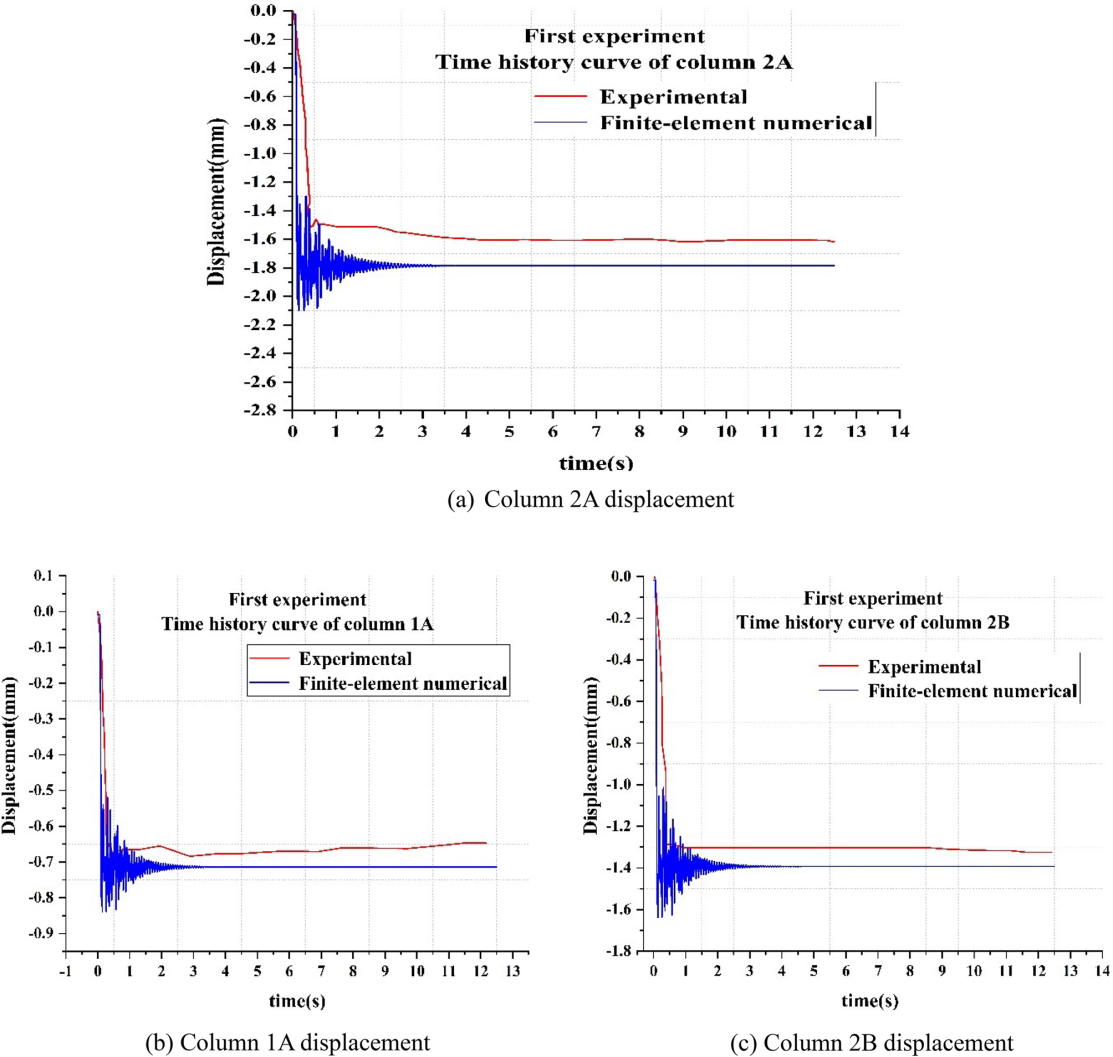
Time history curves of displacement in the first experimentation.

The SAP2000 finite element model is shown in Fig. 18. The time history curve of displacement during the failure condition is shown in Fig. 19a,b,c. From the figure, it is observed that by comparing the experimental value with the finite-element value, the former displacement has no fluctuation section, while the latter displacement has a fluctuation section. Moreover, the overall displacement variation trend of both is the same.

### Second experimental numerical analysis

**Figure 20:**
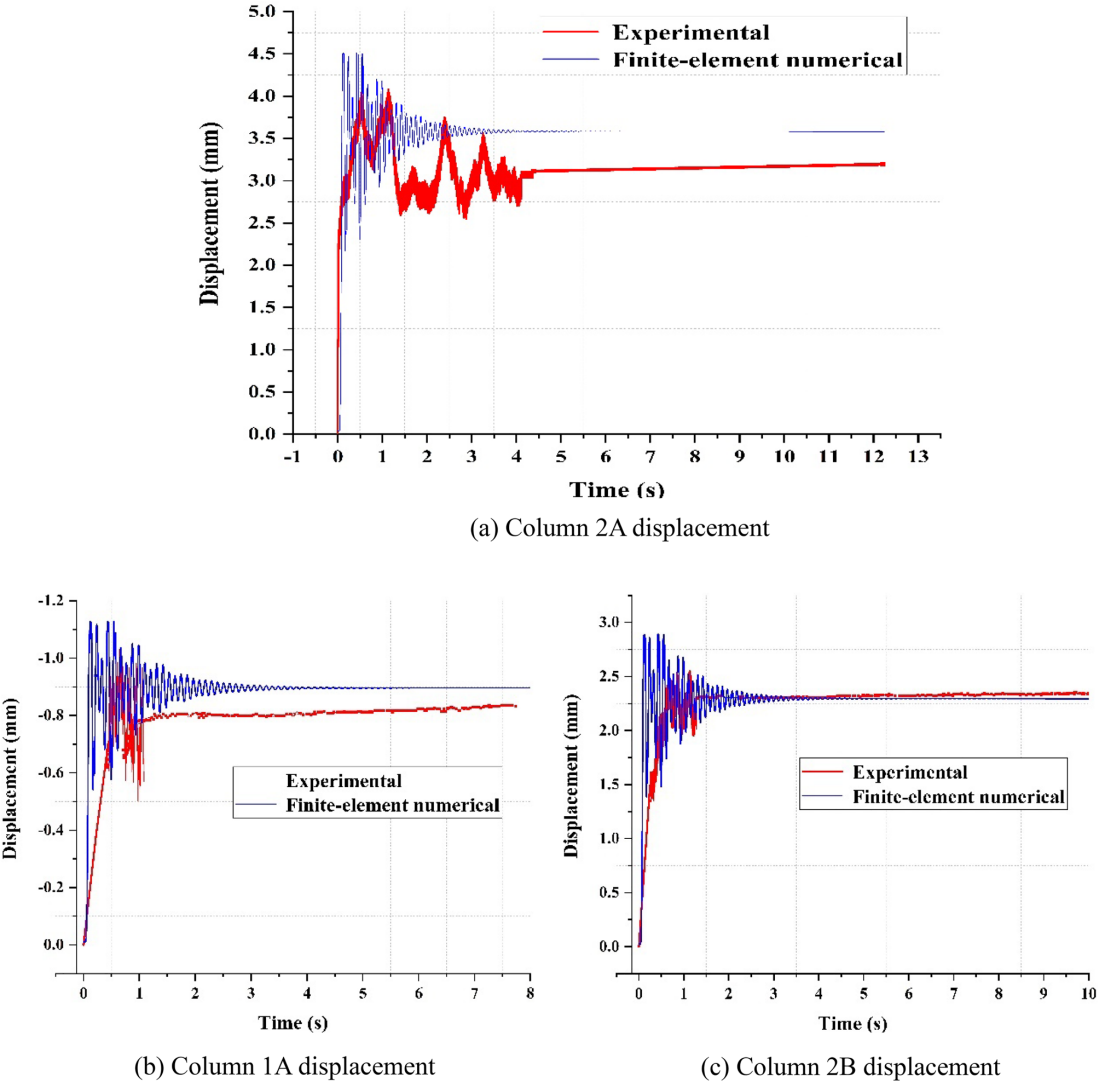
Time history curves of displacement in second experimental.

Under the same failure condition of column A2, the obtained time history curves of displacement are shown in Fig. 20a,b,c. From the figures, it is observed that at the moment of failure, the displacement of joint 10 (8, 11) in X (Y, Z) direction increases quickly and reaches a peak. Then, it was subjected to attenuation vibration until the dynamic force is zero and then the vibration stops. As the E would add to a certain degree under high speed, the finite-element value was found to be slightly larger than the experimental value. However, the general trend is the same, in which E is the elasticity modulus.

### Numerical analysis of collapse ultimate load

**Figure 21:**
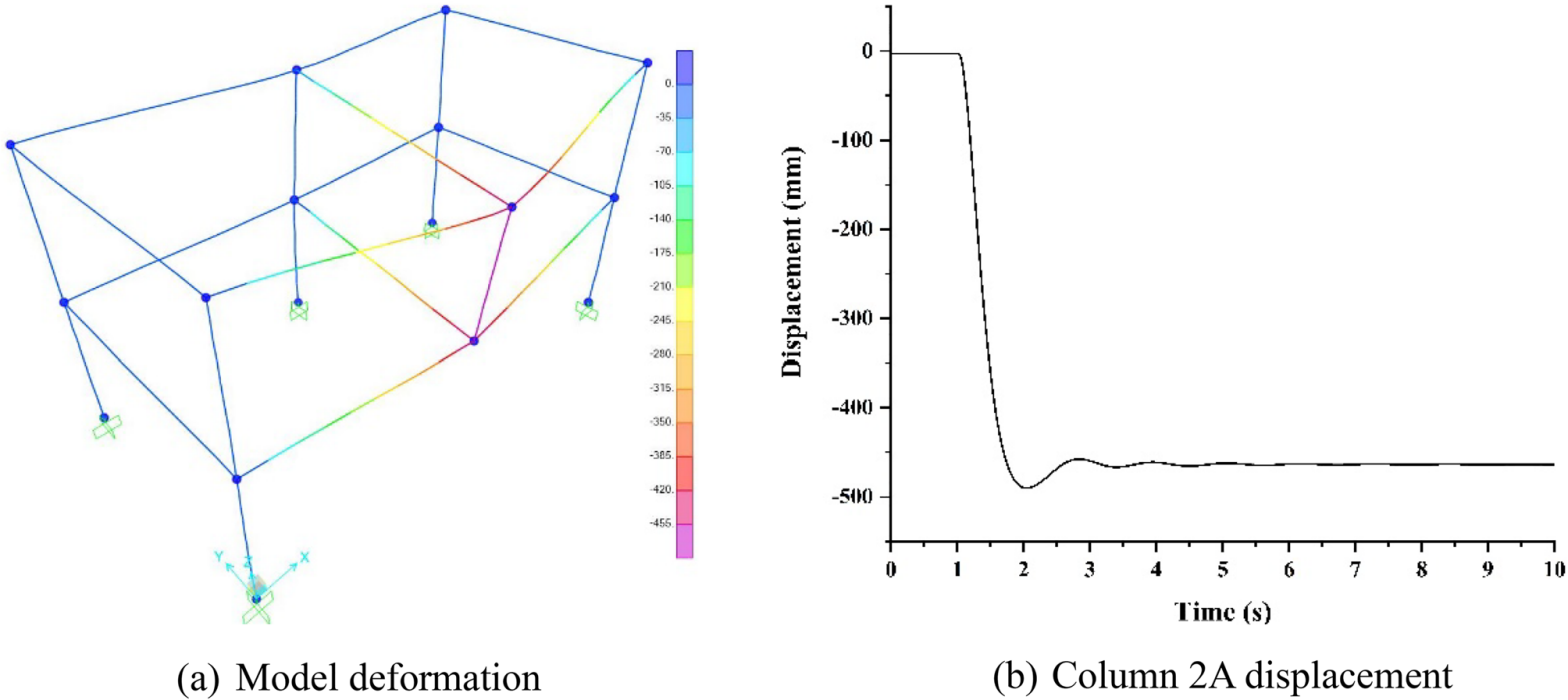
Deformation and displacement under the collapse ultimate load.

The SAP2000 finite element program as per the U.S. Public Affairs Bureau GSA2003, was used to further explore the collapse ultimate load value. The collapse resistance of the RCS structure was evaluated by comparing the collapse ultimate load value with the design load value. It is found that when the line load increases to 334 kN/m on the steel beam, the plastic rotation angle of the horizontal steel beam is 12.76° (the maximum displacement is 489.4 mm), which exceeds the limit specified in GSA2003. Further, the experimental model would progressively collapse. The deformation and displacement under the collapse ultimate load are shown in Fig. 21. From the figure, it is found that the line load was converted into 1500 kN in the most influential area, which was 10.25 times the design load value and far larger than the design load value of the structure. Therefore, it is found that the RCS structure designed with China's building codes^17–19^ was relatively conservative.

## Conclusions

In this paper, it was aimed to explore the collapse ultimate load of the prefabricated RCS composite frame structure, and the “new RCS beam-to-column joint”^15^ was applied to the experimental model. In addition, the complete set of methods for demolition the column 2A was proposed, and the half-scale experimental model (2-story, 1 × 2 bay) was developed. Thereafter, the progressive collapse resistance was studied using the experimental and the numerical analysis. From the results, the following conclusions are drawn:

1. For the progressive collapse resistance experimental of a large space frame structure, the weakened column (2A) was pulled out using a traction force of the vehicle. This method was simple to install, safe, high operability, and had insignificant interference in high-speed data acquisition. The experimental results showed that the method could more practical to respond towards the progressive collapse condition.
2. After the first experimentation, the steel beams connected to column 2A were found to be in the beam mechanism stage, while the remaining RCS structure was in the initial elastic stage. After the second experimentation, the remaining RCS structure was still in the elastic stage.
3. When the load value was 68.4 kN, the displacement time history curve of the RCS structure was different from that of the cast-in-place concrete structure. Specifically, the cast-in-place concrete structure had a displacement vibration phenomenon, while the RCS structure had no displacement vibration phenomenon. When the displacement reached the maximum, the restoring force had to overcome the bolt slip to do work, and the vibration was offset by the friction energy consumption.
4. Moreover, the numerical analysis results were in good agreement with the experimental results, which validated the numerical analysis. SAP2000 was further used to explore the collapse ultimate load of the prefabricated RCS structure, which was found to be 10.25 times the design load. The results showed that the prefabricated RCS composite frame structure designed using the Chinese building codes had better performance on the progressive collapse resistance after the demolition of the single side column.

## Data Availability

The datasets used and/or analyzed during the current study are available from the corresponding author on reasonable request. All data generated during this study are included in this published article.
